# NOD-scid IL2R γ^null^ mice engrafted with human peripheral blood mononuclear cells as a model to test therapeutics targeting human signaling pathways

**DOI:** 10.1186/1479-5876-11-4

**Published:** 2013-01-07

**Authors:** Maryam Zadeh-Khorasani, Thomas Nolte, Thomas D Mueller, Markos Pechlivanis, Franziska Rueff, Andreas Wollenberg, Gert Fricker, Eckhard Wolf, Matthias Siebeck, Roswitha Gropp

**Affiliations:** 1 Department of Surgery, Klinikum der Ludwig-Maximilians Universität München, Munich, 80336, Germany; 2 Institute of Molecular Animal Breeding and Biotechnology, and Laboratory for Functional Genome Analysis (LAFUGA), Gene Center, LMU Munich, Munich, 81377, Germany; 3 Julius von Sachs Institute, University of Würzburg, Würzburg, 87082, Germany; 4 Department of Dermatology, Klinikum der Ludwig-Maximilians Universität München, Munich, 80336, Germany; 5 Institute for Pharmacy and Molecular Biotechnology, University of Heidelberg, Heidelberg, 69120, Germany

## Abstract

**Background:**

Animal models of human inflammatory diseases have limited predictive quality for human clinical trials for various reasons including species specific activation mechanisms and the immunological background of the animals which markedly differs from the genetically heterogeneous and often aged patient population.

**Objective:**

Development of an animal model allowing for testing therapeutics targeting pathways involved in the development of Atopic Dermatitis (AD) with better translatability to the patient.

**Methods:**

NOD-scid IL2R γ^null^ mice engrafted with human peripheral blood mononuclear cells (hPBMC) derived from patients suffering from AD and healthy volunteers were treated with IL-4 and the antagonistic IL-4 variant R121/Y124D (Pitrakinra). Levels of human (h)IgE, amount of B-, T- and plasma- cells and ratio of CD4 : CD8 positive cells served as read out for induction and inhibition of cell proliferation and hIgE secretion. Results were compared to *in vitro* analysis.

**Results:**

hIgE secretion was induced by IL-4 and inhibited by the IL-4 antagonist Pitrakinra *in vivo* when formulated with methylcellulose. B-cells proliferated in response to IL-4 *in vivo*; the effect was abrogated by Pitrakinra. IL-4 shifted CD4 : CD8 ratios *in vitro* and *in vivo* when hPBMC derived from healthy volunteers were used. Pitrakinra reversed the effect. Human PBMC derived from patients with AD remained inert and engrafted mice reflected the individual responses observed *in vitro*.

**Conclusion:**

NOD-scid IL2R γ^null^ mice engrafted with human PBMC reflect the immunological history of the donors and provide a complementary tool to *in vitro* studies. Thus, studies in this model might provide data with better translatability from bench to bedside.

## Introduction

A large number of drug candidates fail in clinical trials due to lack of efficacy and unforeseen toxicity. This is especially relevant in immunological diseases where animal models might not accurately reflect activation mechanisms exerted in humans. The poor predictive quality and translatability of present animal models has been demonstrated in the clinical phase I study of TGN 1412 [1] which tragically triggered a cytokine storm in healthy volunteers despite being well tolerated in cynomolgus monkeys. The shortcomings of present animal models also applies to chronic inflammatory diseases since they mostly rely on the development of the disease in 12-week-old inbred mice maintained under specific pathogen free (SPF) conditions which markedly differ from the pathophysiological mechanisms in the genetically heterogeneous and often aged patient populations [2-4]. Last but not least mouse models cannot be used when protein structures are not sufficiently conserved in mice and humans.

Human peripheral blood mononuclear cells (hPBMC) are widely used for drug screening [5-8], but the example of TGN 1412 has demonstrated that experimental conditions might lead to difficulties in interpretation and translatability of data [9]. Immunological reactions are very often modulated and fine-tuned by temporary, spatial and cell-specific expression of the respective cytokines and their cognate receptors [10,11] and *in vitro* culture conditions may inadequately reflect cell–cell interactions in lymphoid organs in response to an immunological trigger [9].

Therefore, immune-compromised NOD-scid IL2R γ^null^ mice engrafted with human PBMC have become alternative models to study chronic inflammatory diseases such as rheumatoid arthritis [12,13], AD and ulcerative colitis (UC) [14,15]. It has been shown in the AD model, that the immunological background of the donor is crucial to the induction of atopic dermatitis like features and that the immunological imprinting is preserved *in vivo*[14].

AD is a T-helper cell type 2 (Th2) driven inflammation with interleukin-4 (IL-4) and interleukin 13 (IL-13) playing key roles in the early edematous phase [16-18]. IL-4 activates the type I receptor complex consisting of the IL-4 receptor α (IL-4Rα) and the so called common gamma (γc) chain which is thought to be responsible for proliferation and differentiation of T-cells. Both interleukins, IL-4 and IL-13, can also activate the type II interleukin-4 receptor complex consisting of IL-4Rα and the IL-13 receptor α1 (IL-13α1) chain resulting in the generation of phenotypic symptoms such as the immunoglobulin class switch from IgM to IgG_4_ and IgE, fibrosis, epithelial hyperplasia and barrier dysfunction [19-21]. Therefore, both IL-4 receptor complexes have been identified as therapeutic targets. One promising approach is the development of the antagonistic IL-4 variant R121D/Y124D (Pitrakinra), which inhibits the formation and activation of the IL-4 type I and II receptor complexes and has already been tested successfully in clinical phase II studies in asthma [22]. However, limited half-lives of IL-4 or mutant proteins are still a major hurdle in their therapeutic use which results in the need for daily applications of high doses [22-24].

Here we show that studies in NOD-scid IL2R γ^null^ mice engrafted with PBMC complement in vitro studies with cultured hPBMC. IL-4 induced the secretion of IgE *in vitro* and *in vivo* and Pitrakinra abolished the effect of IL-4 as expected. In contrast to previous studies, formulation with methylcellulose was instrumental to the activating and inhibitory effect of IL-4 and Pitrakinra, respectively. The proliferation-inducing effect was not reflected in the spleen of engrafted mice; however, differentiation of T-cells was similar *in vitro* and *in vivo*. Cell batches which responded poorly *in vitro* were inert to IL-4 treatment after transplantation into NOD-scid IL2R γ^null^ mice *in vivo*, indicating that the immunological characteristics of the human donors are preserved in the animal hosts and that results of studies in this humanized model might be better translatable to the patient.

## Material and methods

### Production of wild-type IL-4 and Pitrakinra

Wildtype human IL-4 as well as the IL-4 antagonist/inhibitor Pitrakinra were produced in *E. coli* as described [25]. The amino acid exchanges R121D/Y124D in IL-4 leading to the IL-4 inhibitor known as Pitrakinra were introduced by a two-step PCR mutagenesis and correctness of the cDNA was verified by didesoxy sequencing. For protein production, the cDNA encoding for the mature part of human IL-4 or the variant R121D/Y124D was cloned into the *E. coli* expression vector RBSIIP_N25_x/o [26]. Transformed *E. coli* cells of the strain BL21 (DE3) were grown in LB medium until an optical density of 0.6 to 0.8 at 600 nm was reached. Protein expression was induced by addition of 1 mM IPTG (isopropyl-β-thiogalactosid), expression was continued for a further three hours at 37°C. Cells were harvested by centrifugation and lysed by ultrasonication. IL-4 as well as the variant Pitrakinra were expressed in insoluble form as inclusion bodies, which were dissolved in 20 volumes (v/w) of 6 M guanidinium hydrochloride (GuHCl), 50 mM Tris–HCl pH 8.0. The denatured protein was refolded by a two-step protocol, the first step comprising of a rapid five-fold dilution of the protein solution (protein concentration 2 mg/ml) in 6 M GuHCl into ice-cold water. The solution was stirred for 15 min and then dialyzed against 20 volumes phosphate buffered saline (20 mM sodium phosphate, 120 mM NaCl, 2 mM KCl, pH 7.4) for 24 h at 4°C. The protein solution was buffered to pH 5.5 using 4 M ammonium actetate pH 4.5. Insoluble protein precipitate was removed by centrifugation and the clear supernatant was loaded onto a cation exchange column (GE Healthcare CM sepharose FF). IL-4 was eluted by applying a linear gradient of 0 to 1 M sodium chloride in 20 mM ammonium acetate pH 5.5. IL-4 containing fractions were pooled and subjected to a second purification employing a reversed phase HPLC chromatography. Highly pure IL-4 protein was eluted using a linear gradient of 0.1% trifluoroacetic acid to 100% acetonitrile. Purity was checked by SDS-PAGE and ESI-FT-ICR mass spectrometry. As a quality control, the biological activities and receptor binding properties were examined by measuring the dose dependency of TF-1 cell proliferation for wild-type human IL-4 (EC_50_ of about 10 to 20 pM) as described, or for Pitrakinra by dose-dependent inhibition of the TF-1 cell proliferation [27]. Increasing concentrations of Pitrakinra were added to TF-1 cells, which were simultaneously stimulated with 100 pM wild-type IL-4, the half-maximal concentration of inhibition of TF-1 cell proliferation IC_50_ was determined to be about 5 nM. In addition binding affinity of the recombinant IL-4 proteins (wild-type and the variant Pitrakinra) to its high-affinity receptor IL-4Rα was determined by surface plasmon resonance was described previously [28].

### Isolation and engraftment of human PBMC

Peripheral blood was collected from patients suffering from AD and healthy volunteers. Human PBMC were purified by Ficoll-paque density gradient centrifugation. 30 ml of blood in sodium citrate solution was diluted with 30 ml of HANKS Balanced Salt Solution (Sigma Aldrich, Deisenhofen, Germany) and loaded on Leucosep Tubes (Greiner Bio One, Frickenhausen, Germany). Cells were separated at 800 × g for 15 min at RT according to the manufacturer’s instruction. Human PBMC were isolated, washed with HANKS Balanced Salt Solution supplemented with 2500 IE Heparin Sodium (Braun) and resuspended in phosphate buffered saline (PBS) at a density of 20 × 10^6^ / ml.

Six to 16 weeks old NOD-scid IL2R*γ*^null^ mice were engrafted with 200 μl of the cell suspension (4 × 10^6^ cells) by intravenous injection. The average weight 24.5 ± 4 g. Animals were randomized for age and sex. Following engraftment the animals rested for 7 days.

### Cell culture

4×10^6^ hPBMC were resuspended in 2 ml RPMI, 10% FCS (v/v), 1 mM sodium pyruvate, 10 U Penicillin/Streptomycin, 2 mM Glutamine (Sigma, Deisenhofen, Germany) and incubated for 14 days in a humidified incubator at 37°C and 5% CO_2_ in 24 well plates supplemented with IL-4 (50 ng/ml) and 1 μl anti-CD 40 (1 μg/ml; BD Bioscience, Heidelberg, Germany) as previously described [29,30].

### Study protocol

NOD.cg-Prkdc^scid^ Il2rg^tm1Wjl^/Szj (abbreviated as NOD-scid IL2R γ^null^) mice were obtained from Charles River Laboratories (Sulzfeld, Germany). The mice were housed under specific pathogen free (SPF) conditions in individually ventilated cages. The facility is controlled according to FELASA guidelines. Methylcellulose (A4C Prem) was purchased from Fagron GmbH, Barsbüttel, Germany).

Mice were engrafted on day one. Seven days post engraftment NOD-scid IL2R*γ*^null^ mice (6–16 weeks old) were treated for five consecutive days (day 8–12) intraperitoneally with 10 μg IL-4 dissolved in PBS, or 10 μg IL-4 dissolved in 0.5% methylcellulose (w/v), 0.05% TWEEN 80 (v/v) in PBS or 10 μg IL-4 plus 100 μg Pitrakinra dissolved in 0.5% methylcellulose (w/v), 0.05% (v/v) TWEEN 80 in PBS.

Mice were inspected daily and sacrificed at day 30.

### Serum hIgG and hIgE levels

Samples were blinded and human serum IgG levels were measured turbidimetrically using COBAS INTEGRA 800 (Roche, Penzberg, Germany). Human serum IgE levels were measured by the Elecsys 2010 Immunoassay (Roche, Penzberg, Germany). Murine IgE levels were analyzed by Mouse IgE ELISA kit according to the manufacturer’s instruction (BD Bioscience, Heidelberg, Germany).

### Serum hIL-4 levels

Human serum IL-4 levels were measured using ELISA IL-4 detection kit from BD Biosciences, Heidelberg, Germany at day 10, 24 h post IL-4 administration.

### Flow cytometry analysis

#### Surface phenotyping of human lymphocytes

Labeling of the human lymphocytes, in human and mouse, was performed using the following monoclonal antibodies (mAbs): Anti-human CD3- FITC (clone SK7), anti-human CD4-PE (clone SK3), anti-human CD8-APC (clone SK1), anti-human CD19-APC (clone SJ25C1), anti-human CD38-PE (clone HIT2), anti-human CD45-APC-H7 (clone 2D1), anti-human CD56-PerCP-CY™ 5.5 (clone B159), anti-human CD138-FITC (clone MI15). Anti-mouse CD45 –PE-Cy™ 7 (clone30-F11) staining was performed to exclude all murine host cells from the analysis as well as to check for cross-reactivity with human cells. All mAbs were purchased from BD Biosciences, Heidelberg, Germany.

Human peripheral blood was taken from the vein in heparinized tubes. At the time of sacrifice peripheral mouse blood was collected in heparin coated tubes from the anesthetized mouse by cardiocentesis. 100 μl of blood was stained with appropriate antibodies and incubated for 15 min at room temperature in darkness. The erythrocytes were removed from the samples using 1× BD FACS Lysing Solution (BD Biosciences, Heidelberg, Germany) for 10 min at RT and darkness.

Single cell suspensions were prepared from the spleen in RPMI-1640 containing 10% FCS by mincing with a metal mesh followed by a 100 μm cell strainer (BD Biosciences). The cells were treated by 1× BD Pharm lyse (BD Biosciences, Heidelberg, Germany) for 10 min at RT. Following the washing process in 2% RPMI-1640 (Sigma-Aldrich, Germany), 10^6^ cells/ml were labeled with appropriate antibodies.

Following staining the cells were washed in FACS buffer (PBS containing 2% FCS) and resuspended in 500 μl of the same.

At least 10.000 events were measured using FACS Canto (BD Biosciences, Heidelberg, Germany). Post-acquisition data were analyzed using the FlowJo 7.6.5 software (Tree Star, Ashland, OR).

### Statistical analysis

Statistical analyses were performed using R a free software environment for statistical computing and graphing (http://www.r-project.org). Group means were compared with analysis of variance (ANOVA), followed by Tukey’s test for multiple comparisons. Where assumptions for ANOVA were not fulfilled, Kruskal Wallis test was applied.

### Ethical considerations

All donors gave informed written consent and the study was approved by the IRB of the Medical Faculty at the University of Munich.

All animal studies were approved by the Ethics Committee for animal use of the government of Upper Bavaria, Germany (55.2-1-54-2531-57-10) and performed in compliance with German Animal Welfare Laws.

## Results

### Formulation of IL-4 with methylcellulose induces the secretion of hIgE

**Figure 1:**
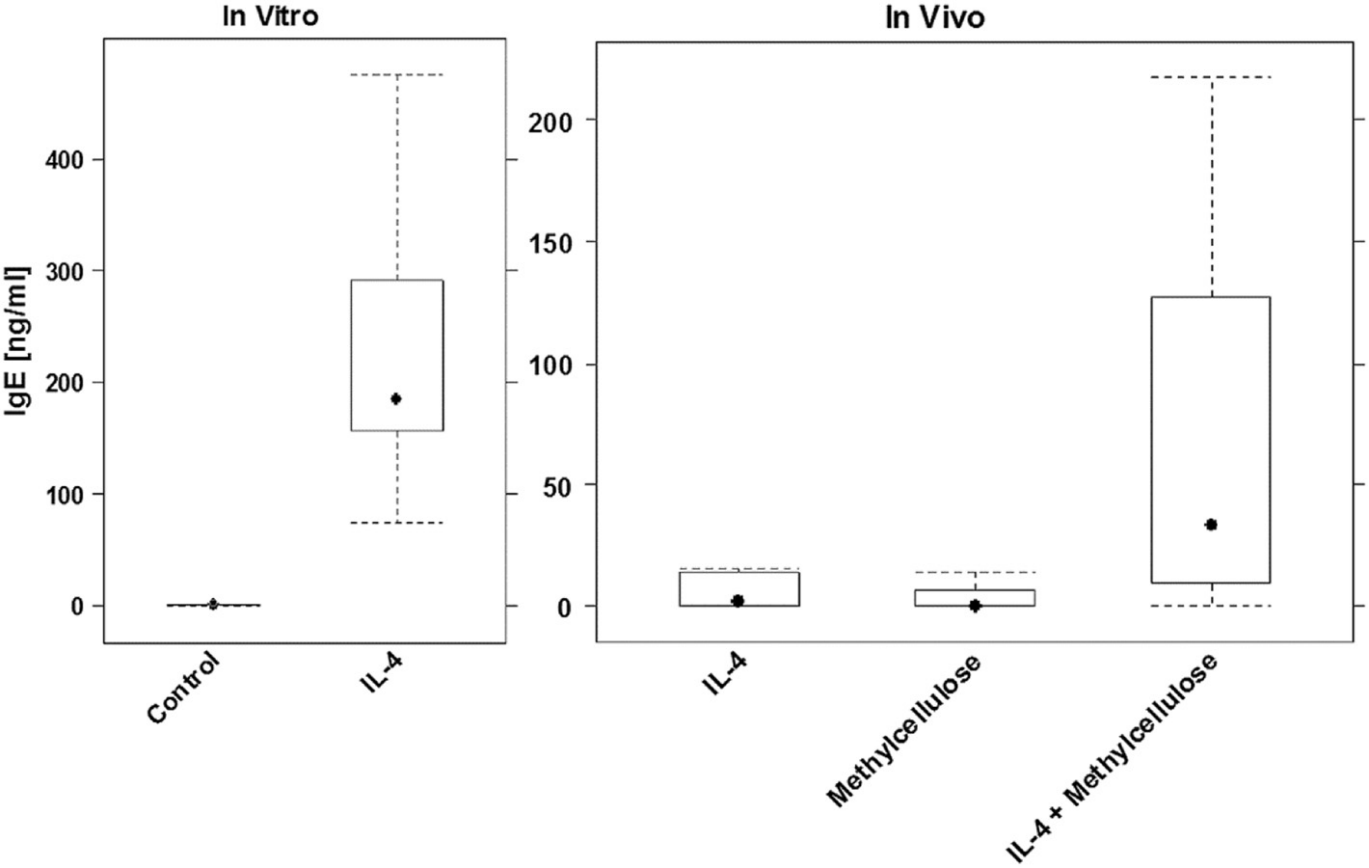
**IL-4 dependent hIgE secretion.** Human PBMC were repeatedly isolated from two different donors and incubated in the absence (control; n = 10) or presence of IL-4 (50 ng/ml; n = 10). After 14 days the hIgE level was determined in the supernatant. For *in vivo* stimulation of hIgE secretion in NOD-scid IL2R γ^null^ mice engrafted with hPBMC from the same donors the animals were treated on five consecutive days (8–12) with 10 μg IL-4, dissolved in PBS (IL-4, n = 10) or 0.5% methylcellulose, 0.05% TWEEN 80 (IL-4 + Methylcellulose; n = 12). Administration of 0.5% methylcellulose, 0.05% TWEEN 80, PBS (Methylcellulose, n = 4) served as control. IgE levels were significantly different in the three *in vivo* groups (Kruskal Wallis test, p = 0.015).

In order to examine whether the formulation of IL-4 with methylcellulose enhances the secretion of hIgE in NOD-scid IL2R γ^null^ mice engrafted with hPBMC, hIgE levels were measured in animals treated with 10 μg IL-4 in PBS (IL-4, n = 10) and animals treated with 10 μg IL-4 formulated with 0.5% methylcellulose, 0.05% TWEEN 80 in PBS (IL-4 + Methylcellulose, n = 12). Engrafted animals treated with carrier alone (Methylcellulose, n = 4) served as control. As we knew from previous experiments [14,15], that the responsiveness to IL-4 is dependent on the immunological background of the donors and their current immunological status, all hPBMC were analyzed simultaneously *in vitro* with respect to their capacity to secrete IgE in response to IL-4. Human PBMC were isolated as described in material and methods and 4 × 10^6^ cells were incubated for 14 days with 50 ng/ml IL-4 as previously described [29]. The induction of hIgE synthesis was measured in the supernatant by immuno assay and turbidimetric measurement. Human PBMC from two volunteers without any symptoms of AD and thus considered as ‘healthy’ responded similarly to IL-4 *in vitro*. Human PBMC from one donor (non-AD1) who had no history of allergy displayed mean values of IgE of 200 ± 94 ng/ml (n = 6) *in vitro*, whereas hPBMC from the other donor (non-AD2) who experienced rare allergic reactions displayed mean IgE values of 275 ± 126 ng/ml (n = 4). Human PBMC of these donors were repeatedly isolated and engrafted into NOD-scid IL2R γ^null^ mice; cohorts consisted of four to six animals. In addition, two donors suffering from AD (AD1, AD2) were included in the study. The isolated hPBMC of both responded upon exposure to IL-4 *in vitro* with the secretion of hIgE (244 and 4248 ng/ml, respectively). Eight days post engraftment mice were treated for five consecutive days as described above according to the treatment regime defined by the respective regime of the group. At day 30 all animals were sacrificed and their blood was analyzed for hIgE levels. As shown in Figure 1 we observed elevated levels only when IL-4 was formulated with methylcellulose. In this group the median level was 33.1 ng/ml as compared to 1.8 ng/ml in the group treated with IL-4 dissolved in PBS and 0 ng/ml in control group treated with carrier alone. The analysis of the data revealed that hIgE levels were significantly different (Kruskal-Wallis, p = 0.013). In this experiment, we observed no difference between the AD and non-AD groups.

### The formulation with methylcellulose prolongs the presence of IL-4 *in vivo*

**Figure 2:**
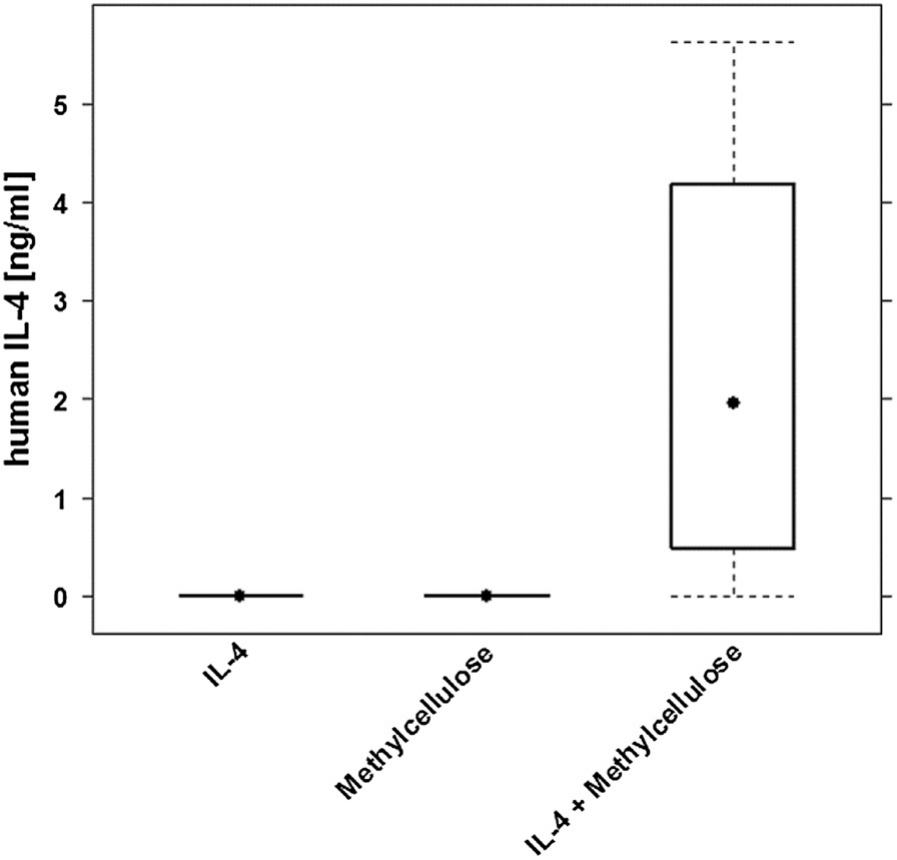
**Formulation dependent IL-4 plasma levels.** Engrafted NOD-scid IL2R γ^null^ mice were treated with 10 μg hIL-4 dissolved in PBS (IL-4; n = 4), 0.5% methylcellulose, 0.05% TWEEN 80 in PBS (Methylcellulose; n = 4) and 10 μg IL-4 formulated with 0.5% methylcellulose, 0.05% TWEEN 80 in PBS (IL-4 + Methylcellulose; n = 11) for five consecutive days (day 8–12). IL-4 serum levels were determined at day 10 24 h post administration. Human IL-4 mouse serum levels in the group “IL-4 + Methylcellulose” were significantly different from the other groups. (ANOVA , F = 4.3, p = 0.03).

In order to examine whether the formulation with methylcellulose results in a prolongation of the half- life of IL-4 *in vivo*, hIL-4 levels were measured in mouse sera as described in material and methods. Blood was drawn at day 9, 24 h after hIL-4 administration in engrafted animals treated with IL-4 dissolved in PBS (n = 4), methylcellulose (n = 4) and IL-4 dissolved in methylcellulose (n = 11). As shown in Figure 2 hIL-4 levels in mouse sera were below detection level in the “IL-4” and “Methylcellulose“ groups and reached a median of 1.95 ng/ml in the “IL-4 + Methylcellulose” group which was significantly different from the other groups. (ANOVA, F = 4.3, p = 0.03).

### The inhibitory effect of the IL-4 variant Pitrakinra on hIgE secretion *in vitro* and *in vivo*

**Figure 3:**
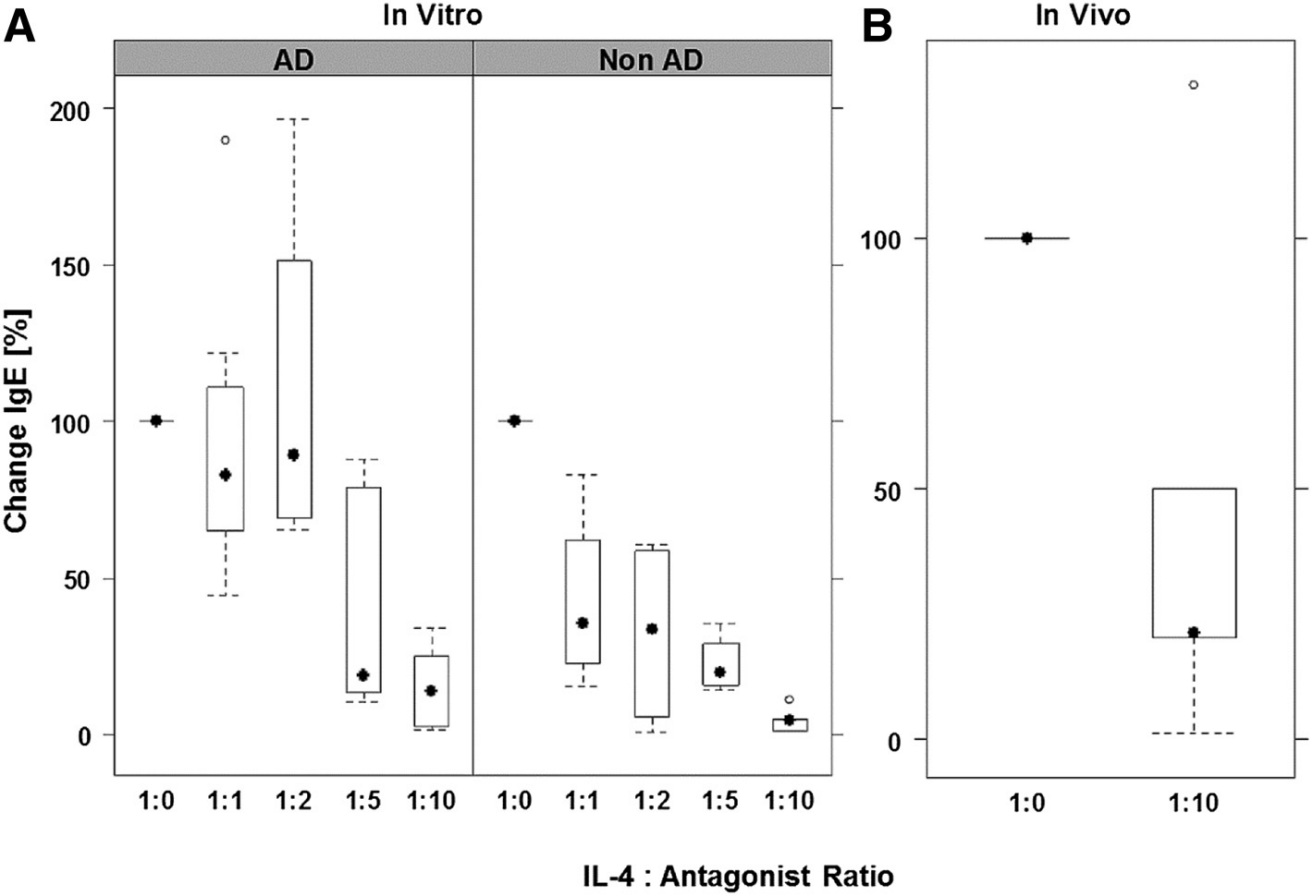
**Antagonist concentration dependent inhibition of hIgE secretion. A**, *in vitro*; Isolated hPBMC derived from patients suffering from AD (n = 5) and from donors without any history of AD (Non-AD, n = 7) were incubated for 14 days in the presence of 50 ng/ml IL-4 and increasing amounts of antagonist (1:1, 50 ng/ml; 1:2, 100 ng/ml; 1:5, 250 ng/ml; 1:10, 500 ng/ml). At a concentration ratio of 1:2 the difference in the AD group was statistically different from the non-AD group (Kruskall-Wallis test, p = 0.02). **B**, *in vivo*; hPBMC derived from patients suffering from AD (n = 4) and from one donor without any history of atopic dermatitis (n = 1) were engrafted and treated with 10 μg IL-4 in 0.5% methylcellulose, 0.05% TWEEN 80 in PBS (1:0) or 10 μg + 100 μg antagonist in 0.5% methylcellulose, 0.05% TWEEN 80 in PBS (1:10). In every cohort (n = 4-5) mean serum hIgE levels were determined. Mean hIgE levels of IL-4 treated animals were defined as 100%. Analysis of the data revealed that the change was not statistically different.

In order to examine whether this animal model might be useful to study inhibitors addressing targets of human inflammatory pathways we analyzed the effect of the IL-4 variant R121D/Y124D (henceforward the variant will be referred to as antagonist or Pitrakinra) in NOD-scid IL2R γ^null^ mice engrafted with hPBMC. To determine the correct dosage of the IL-4 antagonist, hPBMC derived from healthy volunteers and patients suffering from AD were incubated *in vitro* with 50 ng/ml IL-4 in the absence or presence of increasing concentrations of the antagonist (50–500 ng/ml). As in previous experiments hIgE levels were measured by turbidimetry at day 14 with high variability in hIgE levels being seen in both groups upon exposure to IL-4. In order to normalize the inhibitory capacity of the IL-4 antagonist, hIgE values measured in the sample exposed to IL-4 were defined as 100% and the inhibitory effect of antagonist as % change in comparison to the sample treated with IL-4 alone. As shown in Figure 3A secretion of hIgE decreased with increasing antagonist concentration, however, the susceptibility to the antagonist seemed to be different in the AD as compared to the non-AD group. At a concentration of 100 ng / ml antagonist the median change in the AD group was 33.6%, while in the non-AD group the median was 89% and this was statistically significant (Kruskal-Wallis rank sum test, p = 0.02). When the antagonist concentration exceeded the IL-4 concentration by a factor of 10 the remaining hIgE levels were close to zero in both groups.

Therefore, for *in vivo* studies animals were treated with 10 μg IL-4 in methylcellulose or 10 μg IL-4 plus 100 μg Pitrakinra in methylcellulose. Otherwise, treatment regimes were identical to those used in the previous experiments. Animals were engrafted with hPBMC derived from four donors suffering from AD and two without any history of atopic dermatitis. Cohorts consisted of four to six animals. As in the *in vitro* experiments the values were normalized. Mean values of hIgE in the IL-4 treated cohorts were defined as 100% and compared to mean values of the cohorts treated with IL-4 and antagonist. As shown in Figure 3B the median inhibition of hIgE secretion was 22%. The differences between both groups were not statistically significant, but a strong trend was indicated (p = 0.07; Kruskal- Wallis rank sum test).

### FACS analysis of hPBMC and splenic human lymphocytes

In order to analyze to effect of Pitrakinra on T- and B-cells *in vivo* and *in vitro*, hPBMC and isolated human lymphocytes were subjected to FACS analysis. hPBMC isolated from different donors (AD n = 5, non-AD n = 8) were incubated with 50 ng IL-4 or 50 ng IL-4 + 500 ng antagonist as previously described. After 14 days the total numbers of cells were counted in 100 μl of the cell culture.

For the *in vivo* studies animals were engrafted and treated as in the previous study. The AD group consisted of three different donors and the non-AD group consisted of two different donors. The groups were further subdivided into the three different treatment groups: “No treatment”, “IL-4” and “IL-4 + Antagonist” (Table 1).

**Table 1:** Number of animals in the study

**Diagnosis**	**AD**				**Non-AD**			
**Animals**	**Engrafted**	**At the end of experiment**	**Analyzed**	**Δ**	**Engrafted**	**At the end of experiment**	**analyzed**	**Δ**
**Treatment**								
No	4	3	3	1	10	10	8	2
IL-4	18	16	15	3	20	20	14	6
IL-4 + Antagonist	19	18	13	6	10	9	5	5
**Total number**	**41**	**37**	**31**	**10**	**40**	**39**	**27**	**13**

Cohorts consisted of three to six animals. At day 30 the animals were sacrificed and the splenic lymphocytes were isolated as described in material and methods. Engraftment levels were determined by counting of human CD45 positive cells among splenic lymphocytes. In both groups engraftment levels varied significantly and ranged from 0.3 to 71%. There was a strong trend that hPBMC from patients with AD did engraft at higher levels (p = 0.06; Kruskal-Wallis rank sum test). The median engraftment level in the AD group was 16% as compared to the median of 5.5% in the non-AD group. Treatment regimen had no effect on engraftment levels. Animals with engraftment levels below 1% were considered non-engrafted and excluded from the analysis. This criterion applied to six animals of the AD group and 12 of the non-AD group (Table 1).

### The effect of Pitrakinra on B- and plasma-cells *in vitro* and *in vivo*

**Figure 4:**
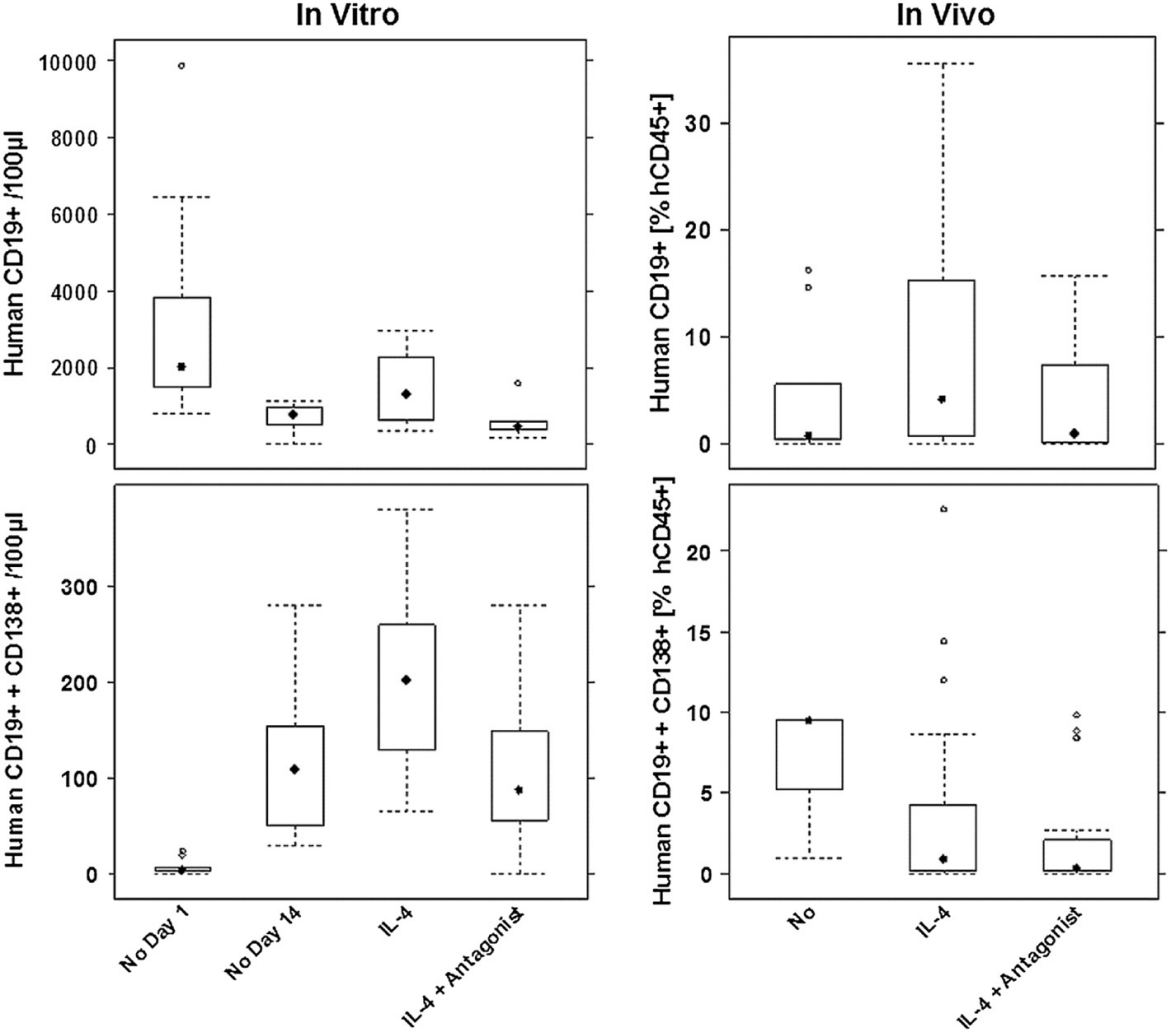
**Effect of antagonist on B- and plasma-cells *in vitro *and *in vivo.****In vitro*: hPBMC (n = 8) were incubated in the presence of 50 ng IL-4 or 50 ng IL-4 + 500 ng Pitrakinra for 14 days. The number of B-cells CD19+ and plasma cells CD19+ + CD 138+ was determined in 100 μl of cell suspensions. Statistical analysis revealed that the difference in Plasma-cells in the “IL-4” group was significant (ANOVA, F = 6.3, p = 0.01). *In vivo*: Mice were engrafted with hPBMC and left without treatment (No, n = 9), treated with 10 μg IL-4 in methylcellulose (n = 18), and IL-4 + Antagonist in methylcellulose (n = 12). The percentage of B- and plasma cells of human lymphocytes was determined. Statistical analysis revealed that the difference in B-cells in the “IL-4” group was significant (Kruskal-Wallis rank sum test, p = 0.03).

As IL-4 is known to induce proliferation and differentiation of B-cells we analyzed the impact of IL-4 and antagonist *in vitro* and *in vivo* by FACS analysis of isolated hPBMC and isolated human splenic lymphocytes from the engrafted animals. For *in vitro* analysis, the number of B-cells (CD19^+^) was determined in 100 μl of the cell suspension by FACS analysis. For *in vivo* analysis, splenic B-cells (CD19^+^) were isolated from the spleen as described in material and methods and the percentage of B-cells was determined by FACS analysis. As shown in Figure 4, treatment with IL-4 resulted in a decline of B-cells after 14 d incubation without any treatment and IL-4 had only a minor effect on total B-cell numbers *in vitro*. In contrast, IL-4 had a pronounced effect on plasma -cells *in vitro* (CD19^+^ + CD138^+^). At day 1 (median = 3) almost no plasma-cells were detectable. The cell culture conditions alone seemed to induce differentiation of plasma-cells as shown by the increase of the median at day 14 (median = 148), an effect which was enhanced by IL-4 (median = 201) and abrogated by antagonist (median = 87). Statistical analysis of log transformed data revealed that the “IL-4” group was significantly different from all the other groups (ANOVA, F = 6.3, p = 0.01).

These results differ from those obtained from the analysis of human lymphocytes recovered from mouse spleens. Here, treatment with 10 μg IL-4 plus methylcellulose resulted in an in increase of B-cells (median = 5.8%) as compared to the non-treated group (median = 1%) and treatment with 10 μg IL-4 plus 100 μg antagonist reduced the percentage of B-cells (median = 0.82%). Statistical analysis revealed that the “IL-4” group was significantly different from the “IL-4 + Antagonist” group (p = 0.05). In contrast, no effect on plasma cells was observed *in vivo*.

### The effect of Pitrakinra on T- cells *in vitro* and *in vivo*

**Figure 5:**
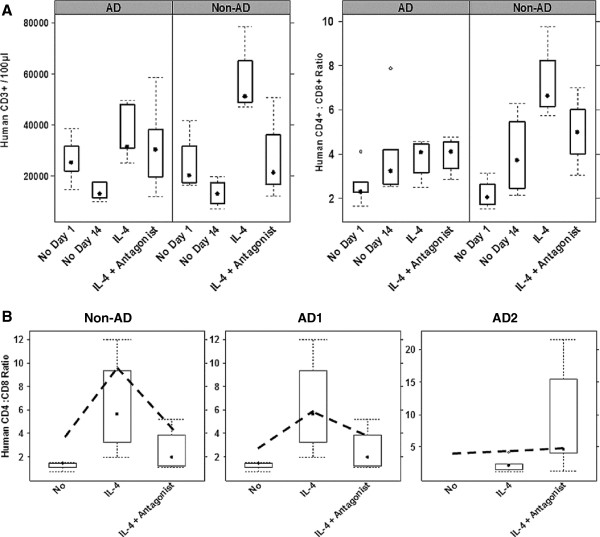
**Impact of IL-4 and antagonist on T-cells *in vitro *and *in vivo. *A**, Relevance of the immunological background on IL-4 induced proliferation of T-cells and differentiation of CD4 cells *in vitro* (AD, n = 7; non-AD, n = 4). Isolated hPBMC were incubated in the absence (No) or presence of 50 ng/ ml IL-4 (IL-4) or 50 ng IL-4 + 500 ng/ml antagonist (IL-4 + Antagonist). **B**, Comparison of individual CD4 : CD8 ratios in response to IL-4 and antagonist *in vitro* and *in vivo*. Non-AD; No treatment (No), n = 6; IL-4, n = 11, IL4 + Antagonist, n = 5), AD1 (No treatment, n = 3; IL-4, n = 4, IL4 + Antagonist, n = 4) and AD2 (IL-4, n = 7; IL4 + Antagonist, n = 5). Dotted lines indicate respective *in vitro* ratios at day 14.

In order to analyze the effect of IL-4 and Pitrakinra on T-cells and subsets of T-cells, human T-cells from the *in vitro* and *in vivo* experiments were subjected to FACS analysis. As shown in Figure 5, IL-4 had a strong proliferation inducing effect on T-cells *in vitro*. The effect was more pronounced in the non-AD group (n = 4), where total numbers increased by a factor of 3.9 (median = 51078) as compared to a factor of 2.8 in the AD-group (n = 7; median = 48040). The difference was not statistically significant. IL-4 and Pitrakinra had no effect on proliferation of T-cells in the spleen of the engrafted mice (data not shown).

As IL-4 is known to influence the ratio of CD4^+^ : CD8^+^ T-cells we further analyzed the ratios *in vitro* and *in vivo*. Although IL-4 induced the proliferation of both, CD4^+^ and CD8^+^ cells (data not shown), the effect was more pronounced in CD4^+^ cells. Unexpectedly, this effect was only observed in the non-AD group. As shown in Figure 5 the ratio in the AD group did not change significantly and was not affected by the antagonist. Mean values were 2.4 ± 0.8 at day 1, slightly increased during the incubation period to 3.6 ± 1.95 at day 14 in the absence of IL-4 and to 4.2 ± 1.16 in the presence of IL-4 and remained at a level of 4.1 ± 0.75 in the presence of antagonist. This was profoundly different in the non-AD group where mean values increased from 2.2 ± 0.6 at day 1 to 3.9 ± 7.2 at day 14 in the absence of IL-4 and significantly increased by a factor of 1.8 to a level of 7.2 ± 1.7. Pitrakinra partially reversed the effect of IL-4 and caused a decrease to a level of 5 ± 2. Statistical analysis revealed that the “IL-4” group was statistically different from all other groups (F = 6.5, p = 0.008 ANOVA multiple comparison).

To test whether these responses were reflected in engrafted animals, we compared the CD4^+^ : CD8^+^ ratios in response to IL-4 and antagonist of individual donors *in vitro* and *in vivo*. The analysis of CD4^+^ : CD8^+^ ratios revealed that the pattern was preserved (Figure 5) whereby the non-AD donor was markedly stimulated by IL-4 (No treatment, n = 6; IL-4, n = 11, IL-4  + Antagonist, n = 5), AD1 moderately stimulated (No treatment, n = 3; IL-4, n = 4, IL4 + Antagonist, n = 4) and AD2 not stimulated (IL-4, n = 7; IL4 + Antagonist, n = 5) by IL-4 and antagonist *in vitro* and *in vivo*.

## Discussion

In this study we describe the development of an animal model that closely reflects the immunological background of the individual human donor and allows for the studying of inhibitors addressing human therapeutic targets involved in chronic inflammatory diseases such as AD *in vivo*. NOD-scid IL2R γ^null^ mice were engrafted with hPBMC derived from patients suffering from AD and from healthy volunteers and treated with IL-4 or the IL-4 antagonist/inhibitor Pitrakinra. We chose IL-4 and its inhibitor Pitrakinra as a model system as the effects of both have been thoroughly characterized *in vitro* and both have been examined in clinical studies [22,24]. A major problem with *in vivo* studies has been the short half-life of IL-4 and Pitrakinra [31,32]. In contrast to previous studies [30,32] we observed no effect when IL-4 was dissolved in PBS which may reflect that in our experiments approximately a tenth of the cell numbers of hPBMC were used for engraftment or the use of different mouse strains. In our studies, hIgE secretion was significantly induced when IL-4 was formulated with methylcellulose, irrespective of the atopic background of the donor. Finally, the IgE secretion reflected the observed induction *in vitro*. The effect was most probably due to an inhibition of the rapid secretion of IL-4 by methylcellulose. In contrast to IL-4 administered with PBS, IL-4 formulated with methylcellulose could be detected in the sera of respective mice 24 h post administration in therapeutic concentrations. However, we cannot conclude from these studies, that long time exposure to IL-4 is also required for priming dendritic cells or T-cells since Biedermann et al. [33] have shown that short time exposure to IL-4 without formulation is sufficient to prime dendritic cells *in vivo*.

Next we analyzed whether the inhibitory effect of Pitrakinra on IgE secretion is reflected in this model. Surprisingly hPBMC derived from AD patients and healthy volunteers responded differently to Pitrakinra *in vitro*. At lower concentration there seemed to be no effect on hIgE secretion in the AD group, while the non-AD group responded to the IL-4 inhibitor. At a ratio of 1: 10 of IL-4 and antagonist both groups responded similarly and the inhibition was also seen in the engrafted animals.

The effect of IL-4 and Pitrakinra on B- and plasma cells produced mirror image results when *in vitro* and *in vivo* experiments were compared, indicating that in this case the environment of the spleen may influence the action and effect of immune-modulatory agents. In the spleen IL-4 induced the proliferation of B-cells and Pitrakinra reversed this effect, while *in vitro* B-cell numbers did not increase. Conversely, plasma cells proliferated *in vitro* in response to IL-4 and Pitrakinra whereas in the spleen no effect of IL-4 and Pitrakinra was detected.

Opposing effects of IL-4 on T-cells were observed *in vitro* and *in vivo* studies. As expected, IL-4 induced significant proliferation of T-cells *in vitro*, an effect that was significantly inhibited by Pitrakinra. In contrast, treatment with IL-4 or Pitrakinra had no effect on engraftment- or T-cell levels in *in vivo* studies.

As expected, IL-4 exerted its capacity to differentiate T-cells *in vitro* and *in vivo* similarly. IL-4 tipped the balance of CD4^+^: CD8^+^ in favor of CD4^+^ cells an effect that was abrogated by Pitrakinra. An interesting and unexpected observation in these experiments was the apparent influence of the immunological background of the donor on the response. PBMC from AD patients hardly responded in *in vitro* studies and individual responses seen *in vitro* were well reflected *in vivo* studies.

Recent studies have shown that high density cell culture of hPBMC mimics cell-cell interactions in lymphoid organs [9]. In our experiments, exposure to IL-4 for 24 h did not prime the cells to produce IgE (data not shown); instead long time incubation was required. The observed individual responses to IL-4 and Pitrakinra indicate that analysis of hPBMC may provide evidence for potential responses in clinical trials. The analysis of the same hPBMC in engrafted mice further allows for detailed studies in lymphoid organs.

In summary, we present an animal model which has the potential to provide data with higher translatability to the patient as compared to conventional mouse models. It complements *in vitro* studies and can be used to stratify patients for later clinical studies.

## Competing interest

The authors declare that they have no competing interests.

## Authors’ contributions

MZK carried out animal studies and FACS analysis. TN carried out animal studies and participated in coordination. TM and MP synthesized IL-4 and Pitrakinra. FR was responsible for patient recruitment and anamnesis. AW participated in FACS analysis. GF formulated the IL-4 and Pitrakinra. EW conceived of the study and participated in writing the manuscript. MS participated in design of the study and the statistical analysis. RG designed the study, analyzed the data and wrote the manuscript. All authors read and approved the final manuscript.
